# Effects of Donor Human Milk and Formula Supplementation on Bone Metabolism and Clinical Outcomes in Preterm Infants Receiving Mother’s Own Milk

**DOI:** 10.3390/nu17203263

**Published:** 2025-10-17

**Authors:** Jacky Herzlich, Bar Frumer, Dror Mandel, Sharon Morag, Ariel Halperin, Laurence Mangel

**Affiliations:** 1 Department of Neonatology, Dana Dwek Children’s Hospital, Tel Aviv Sourasky Medical Center, Tel Aviv 6423906, Israel; 2 Gray Faculty of Medical & Health Sciences, Tel Aviv University, Tel Aviv 6423906, Israel

**Keywords:** preterm infants, donor human milk, preterm formula, bone metabolism, feeding outcomes, necrotizing enterocolitis

## Abstract

**Background:** Human milk (HM) is the optimal nutrition for preterm infants, but supplementation is often required to meet their unique nutritional needs. Donor human milk (DHM) and preterm formula are commonly used alternatives, yet their impacts on bone metabolism and clinical outcomes remain incompletely defined. **Objective:** To compare the effects of exclusive mother’s own milk (MOM), MOM supplemented with DHM, and MOM supplemented with preterm formula on bone metabolism markers, growth milestones, and clinical outcomes in very preterm and very low birth weight (VLBW) infants. **Methods:** We conducted a retrospective review of medical records for infants born at <32 weeks’ gestation or <1500 g birth weight between January 2018 and June 2023. Feeding groups included exclusive MOM (N = 135), MOM + DHM (N = 74), and MOM + Formula (N = 54). Biochemical markers were assessed at baseline and on days 7, 14, and 28. Multivariate regression analyses evaluated predictors of growth and clinical outcomes. **Results:** Infants in the MOM group had significantly lower gestational age and birth weight, with higher rates of respiratory morbidity. Time to full enteral feeding and duration of parenteral nutrition were longer in the MOM group, but feeding regimen was not an independent predictor of these outcomes. By day 14, the MOM group had higher alkaline phosphatase levels and lower phosphorus levels compared to mix feeding groups, but these differences resolved by day 28. Calcium levels varied between groups but remained within normal ranges. Necrotizing Enterocolitis (NEC) incidence did not differ significantly across feeding regimens and was primarily associated with longer parenteral nutrition duration. **Conclusions:** Supplementation of MOM with either DHM or preterm formula supported adequate growth and bone metabolism without increasing NEC risk. Feeding regimen did not independently influence time to full enteral feeding or length of hospitalization, which were driven primarily by infant maturity and clinical status. Both DHM and preterm formula are viable supplements to MOM, ensuring nutritional adequacy without adverse bone health effects. Prospective studies are needed to evaluate long-term outcomes of these feeding strategies.

## 1. Introduction

Human milk is widely regarded as the optimal source of nutrition for preterm infants, providing vital immune protection while supporting growth and development [1,2,3,4]. It is composed of approximately 87% water, 1% protein, 4% lipids, and 7% carbohydrates (including 1–2.4% oligosaccharides), alongside essential minerals and vitamins. However, to meet the unique nutritional requirements of preterm infants and ensure adequate growth and bone mineralization, fortification of human milk is necessary [4,5,6]. Bone mineral deficiency remains a frequent issue in preterm neonates, with prevalence 23–50% for VLBW/ELBW infants and substantial burden carried into childhood [7]. The primary goal is to achieve intrauterine growth rates equivalent to fetuses of the same gestational age and to support optimal neurodevelopment [6,8,9]. Despite advances in neonatal nutrition, achieving optimal postnatal growth in very low birth weight (VLBW) infants remains challenging. Reviews emphasize the importance of early, adequate, and individualized nutrition to minimize extrauterine growth restriction, prioritizing fortified human milk and using specialized formulas when necessary [10,11]. When mother’s own milk (MOM) is unavailable or insufficient, donor human milk (DHM) is the preferred alternative, followed by preterm formula [2,4]. However, DHM differs from MOM due to variations in donor lactation stages and the effects of pasteurization, which reduce its nutritional, immunologic, and bioactive content [12,13]. Formula feeding has been associated with greater weight gain and linear growth compared to DHM but is also linked to an increased risk of necrotizing enterocolitis (NEC) [14,15,16]. NEC remains the most severe neonatal gastrointestinal emergency, associated with significant morbidity, mortality, and adverse neurodevelopmental outcomes, particularly among preterm infants [17].

In addition to gastrointestinal complications, preterm and VLBW infants are at increased risk for reduced bone mineral content (BMC) and metabolic bone disease due to the interruption of third-trimester mineral accretion [18,19]. Adequate calcium, phosphorus, and vitamin D intake is essential to prevent osteopenia and support bone health in this population. Because human milk alone does not provide sufficient minerals for preterm infants, fortification or supplementation with preterm formula is recommended [20,21]. Although DHM offers biological advantages over formula, its use is limited by high costs and the need for specialized infrastructure. Globally, approximately 750 human milk banks exist, predominantly in high-income countries. The World Health Organization recommends DHM as the first alternative when MOM is unavailable or insufficient, particularly for preterm infants born before 32 weeks gestation or with a birth weight below 1500 g [22]. In Israel, the first human milk bank was established in 2019, and our Neonatal Intensive Care Unit (NICU) was among the first to implement routine DHM use in August 2020. Given these considerations, this study aimed to compare the effects of exclusive MOM feeding, MOM supplemented with DHM, and MOM supplemented with preterm formula on bone metabolism markers, growth, and clinical outcomes in very preterm and VLBW infants.

## 2. Methods

### 2.1. Participants

We conducted a retrospective review of medical records for very preterm infants (gestational age [GA] <32 weeks) and VLBW infants (<1500 g) born between 1 January 2018 and 30 June 2023. According to institutional guidelines, these infants were eligible to receive DHM supplementation when indicated, starting from August 2020. Infants included in the analysis were those fed exclusively with MOM, MOM supplemented with DHM, or MOM supplemented with preterm formula (before DHM availability). Both MOM and DHM feeds were fortified in accordance with institutional protocols. Similac Special Care 24 formula was administered during the initial days of life until full enteral feeding of 140 mL/kg/day was achieved, after which infants were transitioned to Similac Neosure formula.

Exclusion criteria were the presence of chromosomal abnormalities, death prior to hospital discharge, or feeding regimens that did not fit the three defined study groups. Biochemical markers of bone metabolism, including alkaline phosphatase (ALKP), phosphorus, and calcium were obtained from the initial blood chemistry panel and subsequently on days 7, 14, and 28 of life. Biochemical markers levels were evaluated using the Siemens ADVIA 2400 Chemistry System based on photometry. This study was approved by the local Institutional Review Board (0172-22-TLV), which granted a waiver of informed consent due to its retrospective design. All procedures were conducted in accordance with Good Clinical Practice guidelines and the Declaration of Helsinki.

### 2.2. Data Collection

Demographic and clinical data collected included GA, sex, birth weight (BW), mode of delivery, use of surfactant, length of hospital stay, and neonatal morbidities (hypoglycemia, respiratory distress syndrome [RDS], NEC, spontaneous intestinal perforation [SIP], bronchopulmonary dysplasia [BPD], feeding intolerance [FI], and bloodstream infection [BSI]). Newborn size classifications—appropriate (AGA), small (SGA), and large for gestational age (LGA)—were defined according to Dollberg’s chart [23]. Growth milestones assessed included the duration of parenteral nutrition (PN), age at initiation of enteral feeding, day of regaining birth weight, and time to achieve full enteral feeding.

NEC was defined as Bell’s stage II or higher. Feeding intolerance was defined as gastric residuals ≥ 20%. Full enteral feeding was defined as achieving an enteral intake ≥ 140 mL/kg/day. Out-of-range bone metabolism markers were defined as ALKP > 500 IU/L, calcium < 7 mg/dL, or phosphorus < 4.8 mg/dL [18,24,25].

### 2.3. Statistical Analysis

Categorical variables were summarized as frequencies and percentages. Continuous variables were assessed for normality using the Shapiro–Wilk test and reported as means ± standard deviations or medians with interquartile ranges, as appropriate. Comparisons of continuous variables between groups were performed using Mann–Whitney U, Kruskal–Wallis, or one-way ANOVA tests. Post hoc analyses were conducted with Bonferroni correction for multiple comparisons, and adjusted *p*-values are reported, as appropriate. Categorical variables were compared using Chi-square or Fisher’s exact tests. Boxplots were used to visually represent the distribution of continuous variables. Multiple linear regression analyses were performed to assess the impact of potential confounders on time to achieve full enteral feeding and length of hospital stay. Logistic regression analyses were conducted to evaluate predictors of NEC and BSI. All analyses were performed using IBM SPSS Statistics for Windows, version 29. A *p*-value < 0.05 was considered statistically significant.

## 3. Results

### 3.1. Study Population

**Figure 1:**
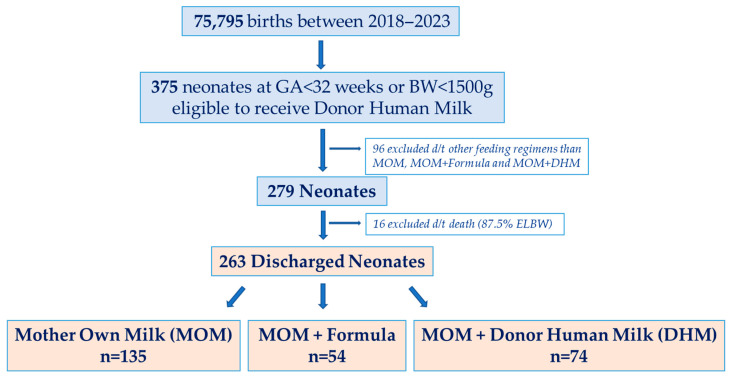
Flowchart of the cohort per feeding regimens.

Of 375 eligible neonates during the six-year study period, 263 were included in the analysis: 135 received exclusive MOM, 54 received MOM supplemented with formula (MOM + Formula), and 74 received MOM supplemented with donor human milk (MOM + DHM) (Figure 1).

### 3.2. Demographic and Feeding Characteristics

Table 1 summarizes demographic and clinical characteristics across feeding groups. Infants in the MOM group had significantly lower GA and BW compared to both mixed feeding groups (*p* < 0.001). Time to initiation of enteral feeding and time to regain BW did not differ significantly among groups.

However, time to achieve full enteral feeding was longer in the MOM group (median 15 days) compared to MOM + Formula (10 days, *p* < 0.001) and MOM + DHM (9 days, *p* < 0.001). Similarly, duration of PN and length of hospital stay were significantly longer in the MOM group. Median hospital stay was 68 days in MOM, compared to 39 days in MOM + Formula (*p* < 0.001) and 43 days in MOM + DHM (*p* = 0.001); MOM + Formula had a significantly shorter stay than MOM + DHM (*p* = 0.020). In mixed feeding groups, MOM constituted the majority of feeds. The median MOM intake percentage was lower in MOM + Formula compared to MOM + DHM (79.5% vs. 90%, *p* = 0.013) (Table 1). Consequently, formula contributed ~20% of total feeds, and DHM ~10%.

### 3.3. Clinical Outcomes

Infants in the MOM group were generally more clinically unstable, with higher rates of RDS and BPD (*p* < 0.001 for both), and greater surfactant use (*p* = 0.002). Hypoglycemia rates were similar across groups (~20%). NEC incidence was higher in MOM group (18.5%) compared to MOM + DHM (12.2%) and MOM + Formula (5.6%), though this did not reach significance (*p* = 0.059). Logistic regression showed only PN duration was independently associated with NEC risk (OR = 1.092 per day, *p* < 0.001), while BW, GA, and feeding regimen were not. BSI rates were higher in the MOM group (20%) compared to MOM + Formula (5.6%) and MOM + DHM (8.1%) (*p* = 0.009). In logistic regression analysis, lower GA (OR = 0.765 per week increase, *p* = 0.014) and longer PN duration (OR = 1.045 per additional day, *p* < 0.001) were significantly associated with increased BSI risk. Feeding regimen was not an independent predictor.

### 3.4. Predictors of Growth and Clinical Outcomes

Duration of PN was longer with lower GA (B = −1.903, *p* = 0.003), lower BW (B = −0.013, *p* = 0.001), FI (B = 6.902, *p* = 0.002), and surfactant use (B = 6.106, *p* = 0.004). Feeding regimen was not predictive (Adjusted R^2^ = 0.320, *p* < 0.001). Time to full enteral feeding was delayed by FI (B = 9.494, *p* < 0.001) and surfactant use (B = 5.842, *p* = 0.003), and reduced with higher BW (B = −0.011 per g, *p* = 0.002) (Adjusted R^2^ = 0.306, *p* < 0.001). Length of hospitalization was shorter with higher GA (B = −6.854, *p* < 0.001) and BW (B = −0.021 per g, *p* < 0.001), but longer with extended PN duration (B = 0.700, *p* < 0.001) and surfactant use (B = 6.682, *p* = 0.006). Feeding regimen showed a non-significant trend (B = 1.606, *p* = 0.059). This model explained 77% of variance (Adjusted R^2^ = 0.768, *p* < 0.001).

### 3.5. Bone Metabolism Markers

Table 2 presents serum bone metabolism markers. ALKP levels were highest in MOM + DHM group on day 7 (median 303 IU/L; MOM [median 261 IU/L] vs. MOM + DHM, adjusted *p* = 0.024). By day 14, MOM had higher ALKP than MOM + Formula (397 vs. 343 IU/L, *p* = 0.005). The proportion with ALKP >500 IU/L was highest in MOM (26.9%) versus MOM + Formula (5.7%) and MOM + DHM (9.7%), *p* < 0.001. Phosphorus levels were lower in MOM on day 14 compared to MOM + Formula (6.1 vs. 6.8 mg/dL, *p* < 0.001) and MOM + DHM (6.1 vs. 6.6 mg/dL, *p* = 0.006), with no difference between the mixed feeding groups. No phosphorus <4.8 mg/dL was observed in MOM + Formula group (*p* = 0.046). Calcium levels varied significantly among groups at all-time points, with consistently lower levels in MOM, though these differences were not clinically significant. No cases of calcium < 7 mg/dL were observed, except one in MOM + DHM at day 14. By day 28, ALKP, phosphorus, and calcium levels were similar across feeding groups.

## 4. Discussion

This study evaluated the impact of three feeding regimens, MOM, MOM supplemented with DHM, and MOM supplemented with formula, on bone metabolism markers, growth milestones, and clinical outcomes in very preterm and VLBW infants. The findings provide important insights into optimal nutritional strategies for this vulnerable population. The composition of MOM evolves with infant growth, rendering it well suited to meet developmental needs [1,2]. DHM has been associated with reduced NEC prevalence; however, its nutritional profile is less optimal due to lower macronutrient levels and nutrient losses during pasteurization [12,13,16]. Gates et al. have shown that DHM has significantly lower levels of protein, sodium, chloride, potassium, and zinc compared to both early and mature preterm human milk [12]. The protein content of DHM may be as little as half the amount found in early preterm milk and substantially below recommended protein intakes for VLBW infants, highlighting the importance of fortification when using DHM to meet nutritional needs [12]. On the other hand, calorie, carbohydrate, calcium, phosphorus, magnesium, and vitamin D content were not statistically different between DHM and preterm milk forms [12]. In contrast, formula provides consistent macronutrient and micronutrient content but lacks critical immunological and cellular components [1,2,15].

Infants in the MOM group had significantly lower GA and BW, reflecting greater illness severity, as evidenced by higher rates of RDS, BPD, and surfactant administration. While time to initiation of enteral feeding and time to regain BW did not differ significantly between groups, the MOM group required a longer time to achieve full enteral feeding and longer durations of PN, resulting in extended hospital stays. Multiple regression analyses confirmed that lower GA and BW, FI, and surfactant use were the primary determinants of prolonged PN duration, delayed full enteral feeding, and extended hospitalization. Feeding regimen itself was not an independent predictor, underscoring that infant maturity and clinical status are more influential than feeding type alone in determining these outcomes.

These observations align with established evidence that preterm birth is the leading cause of neonatal morbidity and mortality worldwide, with risks increasing as GA and BW decrease [26,27,28]. Lower GA and BW are associated with higher rates of respiratory complications and prolonged hospitalizations, emphasizing the need for individualized medical and nutritional management in this population.

Our data also revealed significant differences in bone metabolism markers during the first two weeks of life. By day 14, ALKP levels were highest in the MOM group, with a greater proportion exceeding 500 IU/L, a threshold associated with increased risk of metabolic bone disease [18]. Phosphorus levels were lowest in the MOM group at the same time point, and there was a trend suggesting that MOM + Formula feeding reduced the risk of hypophosphatemia (<4.8 mg/dL). Although calcium levels varied significantly across groups, differences were not clinically meaningful, as almost no cases of hypocalcemia (<7 mg/dL) were observed. Importantly, by day 28, differences in bone metabolism markers across feeding regimens had resolved.

Notably, infants in the mixed feeding groups received predominantly MOM with appropriate fortification, and supplementation with either DHM or formula resulted in bone metabolism marker levels comparable to exclusive MOM feeding at one month of age. These findings align with a prospective cohort study by Körnmann et al., which found no significant differences in bone mineralization or growth at term-corrected age among very preterm infants fed fortified MOM, DHM, or preterm formula, highlighting that adequate fortification is more critical than the milk source itself [29]. Furthermore, our findings showed that predominantly MOM-fed infants supplemented with preterm formula to achieve adequate volume did not exhibit an increased risk of NEC. In our cohort, NEC risk was primarily associated with longer PN duration rather than feeding regimen. Existing evidence suggests that it is the absence of human milk, with its unique immunological and trophic factors, rather than formula exposure per se, that increases NEC risk [30,31,32].

Strengths of this study include its relatively large sample size, detailed biochemical assessments at multiple time points, and rigorous multivariate analyses accounting for key clinical confounders. However, limitations include its retrospective design, which precludes causal inference, and differences in baseline clinical status between groups, which may confound associations despite statistical adjustments.

## 5. Conclusions

In conclusion, supplementation of MOM with either DHM or formula did not delay achievement of full enteral feeding, as delays were primarily attributable to lower BW and greater illness severity in exclusively MOM-fed infants. Additionally, DHM supplementation to predominantly MOM-fed infants resulted in bone metabolism marker levels comparable to those seen with formula supplementation and exclusive MOM feeding by day 28. Formula supplementation, which constituted a median of one-fifth of total intake, was not associated with an increased risk of NEC. These findings suggest that both DHM and preterm formula are viable supplements to MOM when needed, supporting nutritional adequacy without adverse effects on bone health. Future prospective studies are warranted to evaluate the long-term impacts of these feeding strategies on growth, bone health, and neurodevelopmental outcomes in very preterm infants.

## Figures and Tables

**Table 1:** Comparison of demographic and clinical characteristics of the cohort across feeding groups.

Characteristics	MOM N = 135	MOM + Formula N = 54	MOM + DHM N = 74	*p*-Value
Mode of delivery				0.188
Spontaneous vaginal delivery	40 (29.6)	19 (35.2)	30 (40.5)	
Emergency Cesarean section	94 (69.6)	33 (61.1)	42 (56.8)	
Elective Cesarean section	1 (0.7)	2 (3.7)	2 (2.7)	
Infant of diabetic mothers	10 (7.4)	8 (14.8)	11 (14.9)	0.157
Celestone treatment	99 (73.3)	42 (77.8)	61 (82.4)	0.323
Intrauterine growth restriction (IUGR)	24 (17.8)	14 (25.9)	8 (11)	0.090
Gestational age (GA) (week)	28 [27–30]	30 [29–31]	30 [28–31]	**<0.001**
Birth weight (BW) (g)	1070 [825–1320]	1450 [1157.5–1612.5]	1362.5 [1073.8–1486.3]	**<0.001**
Small for Gestational Age	27 (20)	15 (27.8)	12 (16.2)	0.272
Gender (Female)	75 (55.6)	37 (68.5)	43 (58.1)	0.258
Day of initiating enteral feeding (day)	2 [1–2]	2 [1–2]	2 [1–2]	0.058
Day at reaching BW	10 [7–13.3]	9 [6–11]	9 [7–12]	0.260
Duration of parenteral nutrition (day)	15 [10–29]	9 [4.5–10.5]	9 [6–13]	**<0.001**
Day of reaching full enteral feeding (day)	15 [11–28]	10 [6–12]	9 [6.5–13]	**<0.001**
Length of hospital stay (day)	68 [45–95]	39 [31–46.3]	43 [37–74.5]	**<0.001**
Feeding Intolerance	41 (30.4)	9 (16.7)	14 (18.9)	0.062
Surfactant use	74 (54.8)	17 (31.5)	26 (35.1)	**0.002**
Necrotizing Enterocolitis	25 (18.5)	3 (5.6)	9 (12.2)	0.059
Respiratory Distress Syndrome	126 (93.3)	38 (70.4)	55 (74.3)	**<0.001**
Bronchopulmonary Dysplasia	87 (64.4)	8 (14.8)	26 (35.1)	**<0.001**
Blood Stream Infection	27 (20)	3 (5.6)	6 (8.1)	**0.009**
Retinopathy of Prematurity	15 (11.1)	2 (3.7)	8 (10.8)	0.264
Hypoglycemia	27 (20)	10 (18.5)	16 (21.6)	0.909
Percentage of MOM (%)	100	79.5 * [64.4–90]	90 * [79–93]	0.013 *

Data are expressed as mean ± standard deviation or median [Q1–Q3] or n (%). Abbreviations: MOM: Mother Own Milk; DHM: Donor Human Milk. In bold, significant *p* values for Kruskal–Wallis, ANOVA or Chi-square tests as appropriate. * Mann–Whitney test between mixed feeding group.

**Table 2:** Bone metabolism markers across the feeding groups.

Characteristics	MOM N = 135	MOM + Formula N = 54	MOM + DHM N = 74	*p*-Value
Alkaline Phosphatase (ALKP, IU/L) first measurement	189 [156–235]	191 [162–254]	216.5 [170.8–276.8]	0.056
ALKP (IU/L) on day 7	261 * [216.5–337]	277 [205–351]	303 * [256–378]	0.024 *
ALKP > 500 IU/L on day 7	11 (8.3)	2 (3.8)	3 (4.2)	0.482
ALKP (IU/L) on day 14	397 * [300.8–506]	343 * [269.5–413]	350.5 [262.3–457.0]	0.005 *
ALKP > 500 IU/L on day 14	35 (26.9)	3 (5.7)	7 (9.7)	**<0.001**
ALKP (IU/L) on day 28	354.5 [285–473.8]	386.5 [279.8–457.0]	349 [289.5–420.0]	0.609
ALKP > 500 IU/L on day 28	23 (18)	8 (17.4)	7 (10.1)	0.331
Phosphorus (mg/dL) first measurement	5.8 [4.9–6.6]	5.8 [4.9–6.5]	5.5 [4.8–6.4]	0.796
Phosphorus (mg/dL) on day 7	5.7 [4.8–6.6]	6 [5.4–6.9]	5.8 [5.0–6.7]	0.145
Phosphorus < 4.8 mg/dL on day 7	32 (24.2)	5 (10)	13 (18.8)	0.096
Phosphorus (mg/dL) on day 14	6.1 ± 1.1 (3.0–8.4)	6.8 ± 1 (4.9–8.9)	6.6 ± 1.1 (3.3–8.8)	**<0.001**
Phosphorus < 4.8 mg/dL on day 14	12 (9.7)	0 (0)	4 (6.1)	**0.046**
Phosphorus (mg/dL) on day 28	6.4 ± 0.8 (4.2–8.0)	6.6 ± 0.6 (5.5–8.1)	6.6 ± 0.8 (4.4–8.1)	0.218
Phosphorus < 4.8 mg/dL on day 28	5 (4.0)	0	1 (1.5)	0.341
Calcium (mg/dL) first measurement	8.7 ± 0.8 (6.9–10.4)	9.0 ± 0.8 (7.8–11.1)	9.1 ± 0.9 (6.7–11.6)	**<0.001**
Calcium (mg/dL) on day 7	9.9 [9.3–10.3]	10.3 [9.8–10.7]	10.3 [9.8–10.7]	**<0.001**
Calcium < 7 mg/dL on day 7	0	0	0	NA
Calcium (mg/dL) on day 14	10.0 [9.7–10.5]	10.4 [10.1–10.7]	10.4 [10.0–10.8]	**<0.001**
Calcium < 7 mg/dL on day 14	0	0	1 (1.5)	0.483
Calcium (mg/dL) on day 28	10.2 * [9.8–10.5]	10.2 [9.9–10.5]	10.4 * [10.0–10.8]	0.033 *
Calcium < 7 mg/dL on day 28	0	0	0	NA

Data are expressed as mean ± standard deviation or median [Q1–Q3] or n (%). Abbreviations: MOM: Mother Own Milk; DHM: Donor Human Milk. In bold, significant *p* values for Kruskal–Wallis, ANOVA or Chi-square tests as appropriate, * Adjusted *p* value following Bonferroni correction for pairwise comparisons.

## Data Availability

The data presented in this study are available on request from the corresponding author due to privacy.
